# Gene Therapy for Duchenne Muscular Dystrophy: Unlocking the Opportunities in Countries in the Middle East and Beyond

**DOI:** 10.3233/JND-221528

**Published:** 2022-11-08

**Authors:** Haitham Elbashir, Waseem Fathalla, Vivek Mundada, Mehtab Iqbal, Asmaa A Al Tawari, Saleel Chandratre, Laila Bastaki, Ingy Romany, Omar Ismayl, Ahmad Abou Tayoun

**Affiliations:** a Neurosciences Center of Excellence, Al Jalila Children’s Hospital, Dubai, United Arab Emirates; b Sheikh Shakhbout Medical City, Abu Dhabi, United Arab Emirates; c Medcare Women and Children Hospital, Dubai, United Arab Emirates; d Tawam Hospital, Al Ain, Abu Dhabi, United Arab Emirates; e Al Sabah Hospital, Kuwait; f Sheikh Khalifa Medical City, Ajman, United Arab Emirates; g Kuwait Medical Genetic Centre, Kuwait; h Pfizer Gulf FZ LLC, Dubai, United Arab Emirates; i Sheikh Khalifa Medical City, Abu Dhabi, United Arab Emirates; j Genomic Center of Excellence, Al Jalila Children’s Hospital, Dubai, United Arab Emirates

**Keywords:** Duchenne muscular dystrophy, genetic disorders, multidisciplinary care, muscle degeneration, gene therapy

## Abstract

**Background:**

Duchenne muscular dystrophy (DMD) is a severe neuromuscular disorder which leads to progressive muscle degeneration and weakness. Most patients die from cardiac or respiratory failure. Gene transfer therapy offers a promising approach to treating this disorder.

**Objective:**

Given the genetic disease burden, family size, and the high consanguinity rates in the Middle East, our objective is to address current practices and challenges of DMD patient care within two countries in this region, namely the United Arab Emirates and Kuwait, and to outline readiness for gene therapy.

**Methods:**

An expert panel meeting was held to discuss the DMD patient journey, disease awareness, current management of DMD, challenges faced and recommendations for improvement. Opportunities and challenges for gene therapy in both countries were also deliberated. A pre-meeting survey was conducted, and the results were used to guide the discussion during the meeting.

**Results:**

DMD awareness is poor resulting in a delay in referral and diagnosis of patients. Awareness and education initiatives, along with an interconnected referral system could improve early diagnosis. Genetic testing is available in both countries although coverage varies. Corticosteroid therapy is the standard of care however there is often a delay in treatment initiation. Patients with DMD should be diagnosed and managed by a multi-disciplinary team in centers of excellence for neuromuscular disorders. Key success factors to support the introduction of gene therapy include education and training, timely and accessible genetic testing and resolution of reimbursement and cost issues.

**Conclusion:**

There are many challenges facing the management of DMD patients in the United Arab Emirates and Kuwait and most likely other countries within the Middle East. Successful introduction of gene therapy to treat DMD will require careful planning, education, capacity building and prioritization of core initiatives.

## INTRODUCTION

Duchenne muscular dystrophy (DMD) (OMIM #300377) is a severe neuromuscular disorder (NMD) caused by pathogenic variants in the dystrophin gene which lead to progressive muscle degeneration and weakness [1]. The pooled global DMD prevalence is 7.1 cases (95% CI: 5.0–10.1) per 100 000 males and 2.8 cases (95% CI: 1.6–4.6) per 100 000 in the general population, while the pooled global DMD birth prevalence was 19.8 (95% CI:16.6–23.6) per 100 000 live male births [2].

Patients usually present between the ages of 2 and 4 years with early symptoms such as difficulty climbing stairs, a waddling gait and frequent falls [1]. The heart muscles can be affected as early as the age of 10 years [3] and most patients become wheelchair-bound during the second decade of life [4]. The diagnosis is suspected by a high Creatine Kinase (CK) level and confirmed by genetic testing. If a clinical diagnosis of DMD is not confirmed with genetic testing, then a muscle biopsy could be performed to test for the presence of dystrophin protein [5].

Despite therapeutic advances over the past 30 years, there is no effective cure for DMD [1, 6]. The focus has been on supportive care and management of complications where treatment with corticosteroids is the standard of care. However, even with optimal care, most patients with DMD die from cardiac or respiratory failure before or during their 30 s [7, 8].

Guidelines for the multidisciplinary care for DMD have been established which address obtaining a genetic diagnosis and managing the various aspects of the disease [5]. In addition, several therapies that aim to restore the lack of dystrophin protein or address secondary pathology, have received regulatory approval and many others are in clinical development [1]. However, many of these therapies, which are selective to specific gene mutations, remain suboptimal [6]. One promising approach for treating this life-threatening disease is gene replacement therapy that offers the opportunity to correct the underlying genetic defect by the introduction of a functional gene [9]. Another future treatment option is gene editing using CRISPR/CAS9 technology [10].

This paper reports the results of a survey and an Advisory Board meeting (held on 13 November 2021 and hereafter referred to as ‘meeting’) among DMD experts to explore the current practices and challenges in DMD patient care in the United Arab Emirates (UAE) and Kuwait and how to prepare for future gene therapy. Information on the prevalence of DMD in the Middle East is relatively outdated [11] however given the large family size and high consanguinity rates within the Middle East, it is expected that this region has outsized contribution to Mendelian genetic disorders, including DMD [12, 13]. It is difficult to estimate DMD incidence and prevalence for several reasons including sporadic genetic testing send out to different countries. This highlights an important knowledge gap where initiatives (such as a patient registry and centralized local genetic testing) are needed to accurately determine DMD incidence and prevalence in the region. Establishment of a national registry could improve this data gap by gathering representative, real-world data which would enable assessment of both short- and long-term patient outcomes.

The UAE, with a population of 9’991’083 people and a crude birth rate of 10 births per 1000 people, has a comprehensive, government-funded health system and a rapidly developing private health sector [14, 15]. The health care system is well-equipped and well-staffed: there are around 70 hospitals and 150 clinics. Healthcare is provided free in government hospitals; both nationals and non-nationals need to pay or rely on insurance for healthcare in private hospitals. Private hospitals outnumber government hospitals and hence the healthcare is skewed towards a private healthcare setting.

## METHODS

A mixed methods approach was adopted whereby quantitative and qualitative data were collected from a written pre-meeting survey (Supplementary Table 1) and the meeting. The qualitative data included descriptive information on the overall care of patients with DMD.

The pre-meeting survey was conducted to elicit information on the challenges faced and recommendations for improvements in the management of DMD and the opportunities for innovative treatments such as gene therapy, based on collective expert opinion and experience.

The pre-meeting survey results (Supplementary Table 1) were used as a discussion guide during the meeting. The meeting was convened virtually to discuss and debate issues relating to the DMD patient demographic and the pathway from referral to diagnosis, in the UAE and Kuwait. The questions addressed different topics listed in Table 1.

**Table 1:** Topics discussed in the meeting

Topic	Discussion points
DMD referral process	Patient pathway and referral process, including patient volumes; age range; sources of referral; motivation for referral; proportion of national versus non-national patients.
Diagnosis of DMD	Diagnostic timelines and tools including ideal age of diagnosis; time to diagnosis; challenges of DMD diagnosis in the UAE and Kuwait and access to diagnostic genetic testing, including funds for this testing.
Current treatment practices	Current treatment of DMD in the UAE and Kuwait, including challenges with current treatment options; availability and access to new treatment options and source of treatment funding.
Multidisciplinary care in DMD	Access to multidisciplinary care, including composition of the multidisciplinary team (MDT) and challenges faced in providing multidisciplinary care.
Patient support in DMD	Transition and patient support, including hurdles transitioning from paediatric to adult care and access to patient association groups in the UAE and Kuwait.
Gene therapy for DMD	Exploring current unmet needs and potential areas for collaboration to facilitate safe introduction of a new gene therapy for DMD.
Neutralizing antibody (NAb) testing	Adeno-associated virus (AAV) NAb testing including logistics; availability; turnaround time; funding and hurdles.
Key success factors for the introduction of gene therapy for DMD	Gene therapy experiences focusing on challenges currently experienced in gene therapy for other NMDs, and best practice and readiness for the introduction of gene therapy for DMD in the UAE and Kuwait
Access to care	Obstacles in access to care for DMD patients.
Patient Education and awareness	Exploring how to improve patient education and awareness in the UAE and Kuwait.
Expert recommendations and conclusions	Gather expert recommendations on improvements in the management of DMD in the UAE and Kuwait, and the opportunities for innovative treatments such as gene therapy.

Kuwait, with a population of 4’328’553 people and a crude birth rate of 13 births per 1000 people, also has a government-funded health system that is free for all nationals [16, 17]. Non-nationals who are resident in Kuwait are entitled to a health insurance card (for which they pay an annual fee) to access the public system. Private healthcare providers also run medical facilities in the country, available to members of their insurance schemes.

Hence, understanding the landscape of DMD patients’ journey in this region will provide opportunities for better care and management of these patients regionally and globally.

The specific objectives of the meeting were as follows:

1 To better understand the current DMD patient journey in the UAE and Kuwait
2 To identify gaps and unmet needs of current DMD clinical practice / management
3 To characterize the challenges and opportunities for DMD gene therapy

The participants are experts (mainly pediatric neurologists and geneticists) directly involved in the regular care of patients with DMD in the UAE and Kuwait and were selected as they are the physicians responsible for running genetic and neuromuscular services. Patients in primary and secondary care levels are referred to these physicians.

The meeting provided a real-life, clinical based platform to gather insights regarding current clinical practice, and to highlight unmet needs and challenges, based on the experience and expertise of the participants. This information is necessary to drive future clinical strategies in the management of DMD patients, with a particular emphasis on a smooth transition to DMD gene therapy.

## OUTCOMES and DISCUSSION

### The Current DMD Patient Journey in the UAE and Kuwait

#### Early signs and symptoms of DMD

In UAE, in most cases, a pediatric neurologist would typically assess a child based on a positive family history or elevated CK level, however there is no formal referral pathway. In Kuwait, any child born with a positive family history of DMD, is referred to a genetic center or to the pediatric neurologist. Children born to families with a positive history of DMD are usually diagnosed much sooner compared to a first case child in a family.

However, for patients who do not have a family history of DMD, signs and symptoms often go unrecognized. This is primarily due to poor awareness by families, primary and secondary care and the delay in referral to a paediatric neurologist.

Parents are often “falsely” reassured by family, friends, and sometimes even by primary healthcare practitioners (HCPs), that developmental delays observed in their child are within the normal spectrum. Mild delays can be ignored for quite some time as parents rarely mention this to the child’s HCP, especially if there are no other symptoms. Non-motor symptoms (e.g., speech or learning impairment) are rarely recognized as red flags for DMD. As such, patients who present initially with mild delays and non-motor symptoms are at risk of getting lost in the system.

Ultimately, many patients consult with a variety of HCPs (general pediatricians, development specialists; behavioral specialists, rheumatologists and orthopedic surgeons) and often undergo unnecessary work-up e.g., X-rays, liver investigations due to elevated transaminases, and prescribed treatment such as ADHD medication. Differential diagnoses (e.g., vitamin D deficiency, orthopedic (waddling gait / tiptoeing / slow to walk) or behavioral issues) often complicate and delay referral for DMD. Investigations such as serum CK are rarely performed which further delays the pathway to diagnosis. Experts estimate an average 1–2-year delay from onset of first symptoms to assessment by a pediatric neurologist.

### DMD patient referral

**Fig. 1:**
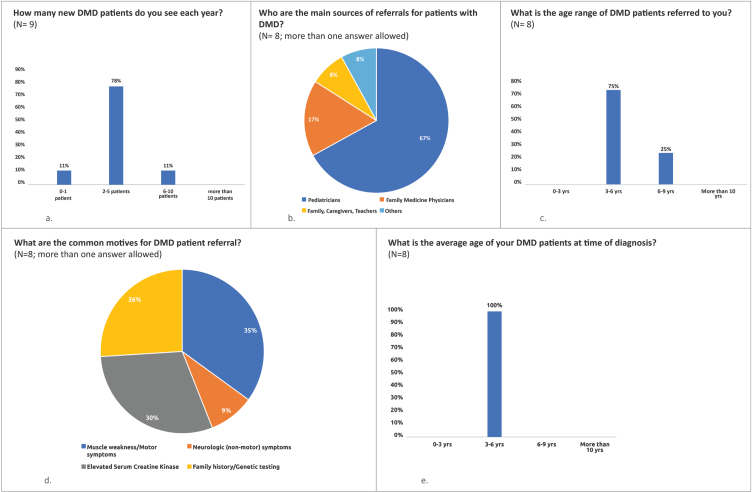
Key pre-meeting survey results. (a) to (e) indicate the percentage of respondents who selected the relevant answer to the question. N = number of respondents.

There continues to be a time delay in DMD referral and diagnosis [18]. In the UAE and Kuwait, experts estimate that HCPs see on average between 2 and 5 new DMD patients per year (Fig. 1a). Children are generally referred (most commonly by pediatricians) to pediatric neurologists between the ages of 3 to 9 years (Fig. 1b and c). Muscle weakness and other motor symptoms are the most common motives for referral (Fig. 1d). Nationals account for 11.5% [19] and 30% [20] of the population in the UAE and Kuwait respectively— the majority of the population in both countries are non-nationals.

In the UAE, there is no fast-track system in place however patients are generally referred within 4-6 weeks to see a pediatric neurologist.

In Kuwait, suspicious cases are fast tracked where patients wait 2–3 weeks for an appointment with a pediatric neurologist. There are satellite clinics in hospitals to ease referral between neurology and genetics with a fast-track system for genetic testing. All genetic testing is done locally, facilitating faster results. There is no difference in the referral pathway for nationals *versus* non-national patients.

In the current therapeutic landscape, although referral delays do not pose a significant impact on patient management, when innovative treatments such as gene therapy become available and early initiation of treatment is vital, timeous referral might become more critical for optimal patient outcomes.

More recently, some trends have begun to reverse in the UAE, and specifically in Dubai with the announcement of the Dubai Academic Health Corporation (DAHC). All public hospitals have merged under one umbrella to facilitate patient transitions, especially between pediatric and adult care, alongside the centralization of patients’ electronic medical records. Furthermore, with the establishment of the CAP-accredited Al Jalila Genomics Center (www.genomics.ae), all genetic testing, including that for DMD, is being performed locally with a faster turnaround time and is often accompanied with appropriate genetic counseling. This new infrastructure will facilitate the early diagnosis and management of patients with DMD, especially given that (as mentioned above) most of these patients are UAE nationals and have free access to healthcare services within the DAHC.

### DMD diagnosis

Currently, experts estimate that the average age of diagnosis of DMD in the UAE and Kuwait is between 3 –6 years, rarely earlier particularly when there is no family history (Fig. 1e).

Notably, the mean age at diagnosis has been reported to be 4.3 years in the UK with a delay in diagnosis averaging 2–3 years. As noted by these authors, earlier diagnosis is likely to result in better opportunities for optimized care [21].

Similarly in the UAE and Kuwait, importance is placed on early diagnosis. Early diagnosis of DMD patients not only enhances the efficacy of any treatment, mainly gene therapies but it can also cut costs by avoiding unnecessary diagnostic workup, multiple visits, and inappropriate treatment/management plans.

The most common and frequently followed screening test in DMD patients is CK testing. CK testing is simple and is recommended to be done routinely at primary, secondary or tertiary level, if DMD is suspected. The test is usually covered by insurance for those patients with access to private hospitals. If CK levels are high, the child should be referred for genetic testing. Muscle biopsy is only necessary if genetic testing is inconclusive. If muscle biopsy is performed, the sample may be sent abroad for detailed analysis as pediatric neuromuscular histopathologists are not readily available.

### Genetic testing in DMD patients

**Fig. 2:**
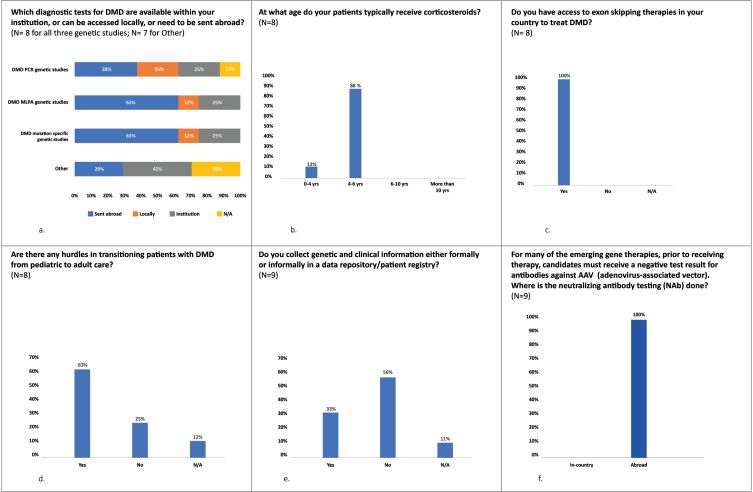
Key pre-meeting survey results continued (a) to (f) indicate the percentage of respondents who selected the relevant answer to the question. N = number of respondents.

In the UAE, genetic testing is conducted free of charge for nationals only however the pathway for reimbursement is changing. While this testing can be performed locally in Dubai, it is generally sent out by most other hospitals outside this Emirate (Fig. 2a). Non-nationals will either self-pay or insurance will cover the cost. In Abu Dhabi, genetic testing is either covered by insurance or patients self-pay. However, in Kuwait, it is conducted locally and is free of charge for both nationals and non-nationals. There are currently 139 DMD patients (  93 Kuwaiti and 46 non-nationals) in Kuwait. Available genetic testing includes fragment analysis mainly by Multiplex-ligation dependent probe amplification (MLPA) for deletion and duplication analysis, and next generation sequencing (NGS) testing, in the form of targeted neuromuscular panels and whole exome sequencing, to capture both sequence and copy number variants (usually through sophisticated depth of coverage algorithms) [22–24]. MLPA is conducted first and if negative (with strong clinical suspicion of DMD), next-generation sequencing/whole exome sequencing is performed. It takes between 4–12 weeks for the results of the genetic testing to be received, depending on the test requested.

### Current Management of DMD in the UAE and Kuwait

Patients typically receive corticosteroids between the ages of 4 and 6 years (Fig. 2b). In the UAE, Deflazacort is ordinarily prescribed first, followed by prednisone (albeit many hospitals do not have Deflazacort). In Kuwait, only prednisone is available. The majority of DMD patients return for follow up / monitoring visits every 3 –6 months.

In the UAE and Kuwait, new medications for DMD are becoming available including exon skipping medications (Fig. 2c).

Although there have been some efforts to publish regional guidelines on managing DMD patients, which take into consideration peculiarities of every healthcare system, there is currently no need to develop new clinical guidelines. Nonetheless, when gene therapy becomes available, new guidelines might be needed to assist HCPs in navigating the available therapeutic options in the context of each patient’s profile.

### Multidisciplinary care of DMD patients

DMD care is demanding and requires input from many HCPs. In the UAE and Kuwait, the primary HCPs that form part of the MDT include pediatric neurologists with neuromuscular expertise; physiotherapists/rehabilitation therapists; cardiologists; pulmonologists; dieticians and genetic counsellors. Spinal surgeons, orthopaedic surgeons and clinical psychologists are also part of the wider MDT.

In the UAE, DMD care can be fragmented. Services are not consistently provided in one single hospital and there is generally no structured communication between facilities. However, as mentioned above, with the transitioning into an interconnected academic healthcare system in Dubai, patients will have a more streamlined journey starting with fast diagnostics, neurology referral, and smooth transitioning between pediatric and adult care given the centralized electronic medical records within this system.

In Kuwait, MDTs are localized in each hospital but not consistent across hospitals. There are future plans to establish a new center, offering a centralized DMD MDT clinic which will lead to improved DMD patient management. A committee has been established in the Ministry of Health (MOH) for all new gene therapy treatments, which might facilitate multidisciplinary care for DMD patients.

Sustainability and continuity of multidisciplinary care is challenging with limited support in the community / local clinics, e.g., physiotherapy sessions are conducted at tertiary clinics. The availability of multidisciplinary care services is different depending on insurance approvals.

Ideally, the future management of DMD patients should be based on a hub-and-spoke model between centers of excellence for NMD with access to local genetic testing, and local centers with expertise in NMD offering care closer to home.

### Transition to adult care

There are several hurdles in transitioning patients with DMD from pediatric to adult care, including a lack of services for adults, patients’ attachment to pediatric neurologists and a lack of transitional clinics. Patients are typically treated in children’s hospitals, which necessitates a referral to a general hospital. Based on our survey, the majority of experts experience difficulties in transitioning patients from pediatric to adult care (Fig. 2d).

In Dubai, the transition will be significantly mitigated with the new centralized healthcare system which will connect all public, pediatric and adult hospitals under one umbrella.

In Kuwait, experts advise that the average referral age of patients with DMD is 12 years. There is a smooth transition from pediatric to adult care with continuity of services provided by adult neurologists with experience in treating DMD in adults. Informal patient support groups are available, and parents also make collective efforts in their private capacity to support their children.

### Screening, research and DMD patient registries

In the UAE, the national new-born screening program does not include testing for DMD. In Kuwait, the Kuwait Genetic Center offers screening for other NMD but not for DMD.

A major obstacle in understanding DMD disease attributes in the Middle East region, i.e., its prevalence, incidence, and the pathogenic variant spectrum, is the lack of methodologically sound and large-scale epidemiological studies [4]. There is a need for patient registries and data management. As expected, our survey shows that 56% of the DMD experts do not locally collect clinical and genetic data about their DMD patients (Fig. 2e). Most data are based on personal experiences of clinicians. Furthermore, the UAE has a fragmented healthcare system and genetic testing is sporadic: different hospitals send out genetic tests to different laboratories in Europe, US, India, etc. This highlights an important knowledge gap which needs to be addressed in the UAE. In Kuwait, however, there is a centralized system which allows for data collection.

It is estimated that the number of newly diagnosed cases of DMD is between 2-3 cases per 10000 births annually.

### Key Challenges and Recommendations in the Management of DMD in the UAE and Kuwait

The key challenges and recommendations on how to improve DMD management are listed in Table 2.

**Table 2:** Key challenges and recommendations in the management of DMD in the UAE and Kuwait

Key challenges	Recommendations
Suspicion of DMD	The awareness issues for DMD referral are similar for the UAE and Kuwait, and among nationals and non-nationals.	There is a great need for awareness and educational campaigns, particularly pertaining to the signs and symptoms of DMD. These initiatives should target both the medical community (general practitioners and paediatricians) and the public to help improve early diagnosis. Awareness and education of DMD suspicion as well as standardizing the North Star Ambulatory Assessment (NSAA) among physiatrists and physiotherapists is important in enabling them to identify the early signs and symptoms of the disease and to monitor the progression of the disease and treatment effects. Example of awareness campaigns include Neuromuscular Study Days for MDT professionals and Neuromuscular Family Days for patients, their siblings, and carers. These initiatives highlight the disease in both the medical community and general public.
DMD referral process	In the UAE, the referral pathway is complicated by a divided private/public healthcare system, with no standard DMD patient referral pathway. In addition, there is a lack of a centralized system for medical records to track patients. In the private sector there are several hospitals, with no intercommunication between different facilities, leading to some patients getting lost in the system. In many cases, the hospitals follow an insurance-based system which can result in a delay in referral due the approval process. This impacts on access to secondary and tertiary care especially for non-nationals with low insurance coverage, as patients with lower coverage typically receive care at a primary level clinic before being referred to secondary and tertiary hospitals.	There is a need to establish a strong, interconnected referral system between primary, secondary, and tertiary care hospitals of different health sectors, to bridge gaps between HCPs and to avoid delays in diagnosing and referring DMD patients. Networking between hospitals and satellite clinics in hospitals can facilitate streamlined referral between neurology and genetics. Raising awareness in the community (among parents and clinicians) can also help to improve parents’ understanding of the disease to decrease existing delays in referrals. Provide training to insurance companies on the importance of early referral of patients suspected to have DMD (or patients with a high CK level) from primary to secondary level of care, irrespective of their insurance coverage.
Diagnosis of DMD	The average wait time between referral and diagnosis in the UAE and Kuwait could be up to 1 year, with most patients visiting many HCPs before receiving the correct diagnosis.	With increased awareness, newborn screening and earlier intervention, DMD cases could be detected before the onset of symptoms which would likely result in a better prognosis. The level of suspicion of DMD should be increased for boys where there are delays in speech, other developmental milestones or abnormal behavior. Even though it is often asymptomatic, the disease process starts early, and it is important to implement support measures, i.e., discussions around nutrition, bone health, rehabilitation, treatment with corticosteroids (or exon skipping therapy) etc., even before the patient becomes symptomatic to improve the prognosis.
Genetic testing in DMD	In the UAE, there is no universal coverage for genetic testing: it is covered by the government for nationals only but not for non-nationals however the reimbursement pathway is changing. Patients without private insurance will have difficulty in receiving genetic testing and they will have to self-pay to get tested. In Kuwait, genetic testing is covered for both nationals and non-nationals.	Pharmaceutical companies could support genetic testing for patients either through supporting local laboratories to conduct the genetic testing or covering the cost of doing the test abroad.
Current management of DMD	The resultant rise in Body Mass Index (BMI) that DMD patients experience due to corticosteroid use poses a challenge. The conversation about when to initiate corticosteroid therapy with parents is a controversial issue: parents are often reluctant to initiate their child on corticosteroid treatment early due to a perceived imbalance between side effects and efficacy. This, together with delayed referral and diagnosis, causes a delay in the initiation of corticosteroid therapy. There are children aged 7 – 8 years in Dubai clinics who are still not on corticosteroids.	Early discussion with parents regarding corticosteroids is important. These should highlight the long-term treatment benefits in terms of ambulation and long-term benefits on cardio-respiratory functions. Paediatric neurologists with experience in treating and managing NMD can form a network to offer peer support and second opinions to families who are undecided. Different treatment regimens / formulations should be discussed if a child experiences side effects. Information leaflets highlighting the benefits and potential side effects (supported by evidence from published trials) of corticosteroids should be provided, ideally in the local language. Early diagnosis of DMD patients is very important as it can: • cut costs by avoiding unnecessary diagnostic workup, multiple visits, inappropriate treatment/management plans • enhance the efficacy of treatment (mainly gene therapies) if delivered as early as possible and improve patient outcomes • provide an option for a patient to participate in a clinical trial and guide families to make informed decisions Pilot early screening programs (genetic screening) and patient registries are needed to generate the relevant, reliable data to formulate reimbursement arguments to guide payers in their reimbursement decisions. The denial of effective gene-based therapies does not necessarily save the healthcare system money as the financial burden of the healthcare of untreated DMD patients can outweigh the costs of gene-based therapies. Collaboration between Pharma, Charitable Organizations, Ministries and insurance companies is essential to develop different economic models to ensure access of high-cost medications to patients.
Multidisciplinary care in DMD	There are insufficient personnel to support the MDT. Where there are sufficient personnel, it is often a challenge for all the HCPs to work as a team in one clinic at the same time: a patient may be seen by all the necessary physicians but not always in the same hospital nor on the same day. Insurance coverage limits the opportunity for a MDT clinic, as only one HCP’s fee is usually covered. This implies that a patient must make separate appointments with the different HCPs, with varying wait-times for each. Furthermore, insurance dictates the extent of treatment (and genetic testing), i.e., only a certain number of rehabilitation sessions might be covered.	Ideally, patients with DMD should be managed in centers of excellence for NMD. As such, patients would be treated in one facility by a dedicated MDT. Centers of excellence for DMD patient care would offer advantages for both patients and physicians, as it will support easy access to treatment and a dedicated, well-trained MDT. Dedicated centers would reduce variability and instill standard management protocols and care pathways across the centers so that all patients with DMD would receive the same care irrespective of insurance limitations. These centers could optimize genetic testing and counselling for personalized treatment. In addition, they would facilitate local research by supporting the set-up of patient registries and collection of real-world data on DMD, phenotypes and genetic mutations and enable patient participation in clinical trials.
Patient support in DMD	Patient advocacy groups for NMD in the ME region are limited. In the UAE, there is a Rare Disease Society (https://uaerds.ae/) which is a patient advocacy group for rare diseases however there are no formal patient advocacy groups for DMD specifically. This society has recently started reaching out to patients and experts which is a positive step. In Kuwait, there are informal family groups (driven by individual efforts) however there are no established patient support groups for DMD patients.	Overall, patient advocacy groups can play a significant role in providing awareness and education; emotional support; practical daily living support and advocacy and policy reform. The role of patient advocacy groups in the future will be most important in not only supporting DMD patients and their families but also to better understand their perspectives and unmet needs. Hopefully, this will improve in the future and patient advocacy groups will become available.
Access to care	Cost is a major issue as evolving therapeutics are extremely expensive. In the UAE, nationals who do not have insurance or their insurance provider declines to pay for novel therapies, are covered by the government. This is the case for all currently available SMA treatments including gene therapy and the new DMD treatments (Exon-skipping and non-sense mutation). However, for non-nationals, if their insurance does not cover the treatment, they will have no option but either to self- pay or to reach out to local charities. In other countries, many patients utilize crowd funding options to get the funding required to pay for gene therapy but not for other repeat medications. The high cost of medications, non-covered genetic and inherited conditions and internal reviews by different hierarchy within the insurance company can contribute to the delay in approval which ultimately delays treatment initiation. In Kuwait, the approval of high-cost medication for nationals is managed by the government through an approval committee. All Kuwaiti national patients are funded by the government however it can take up to 4 months for approval to be granted. Health insurance companies do not cover neuromuscular therapies for non-nationals.	Financial models on the economic burden of DMD are needed for reimbursement discussions with health authorities and insurance companies. These should include information on the health economic burden of untreated patients (e.g., cost of long-term care facilities, wheelchair bound patients); the economic advantage of early treatment; indirect cost savings from integration in society/school and examples of how DMD management is financed in other countries.

### Opportunities and Challenges for Gene Therapy in DMD

Over the past 10 years, there has been a growing interest in innovative therapeutic approaches to treating DMD. In recent years, an increasing number of therapies have been studied which use different genetic techniques [4].

Gene therapy has the potential to provide long-lasting therapy with a single treatment [25]. Currently there are ongoing trials which differ in the type of AAV used, the parts of dystrophin selected for the micro-dystrophin versions, and the gene switch used to ensure proper expression of the micro-dystrophin in skeletal muscle and the heart [26, 27]. Compared with other therapies, AAV gene therapy shows the most promising success so far— it also has the potential of treating a large proportion of patients with DMD [6]. Other therapies such as exon skipping and stop codon readthrough may also be beneficial, although these therapies are selective to specific gene mutations and as such would only be applicable to a smaller proportion of DMD patients [6].

### Key Success Factors for the Introduction of DMD Gene Therapy

**Fig. 3:**
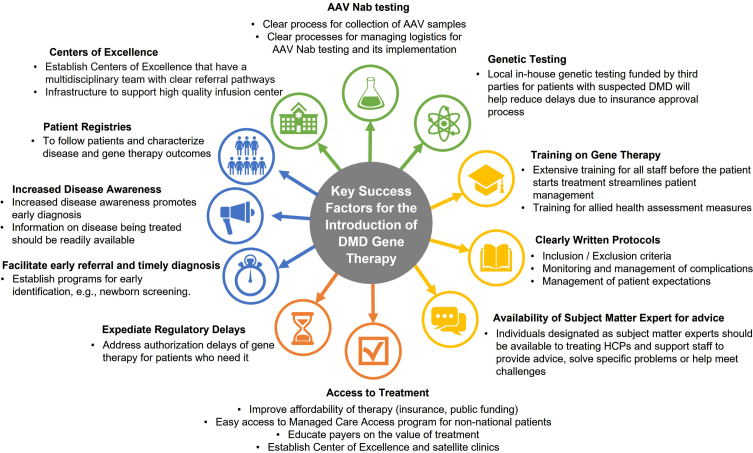
Key success factors for the introduction of DMD gene therapy.

Figure 3 illustrates the key success factors for introducing DMD gene therapy in the UAE and Kuwait. These factors were discussed in the meeting and are based on challenges and recommendations pertaining to expert experience and learnings in providing SMA gene therapy.

NAb testing provides insight into the safety and efficacy of biologic therapies by assessing potential drug inhibition or neutralization through the production of anti-drug antibodies. As such, testing for NAb against AAV is an integral assessment that is conducted for eligibility prior to initial gene therapy.

In general, AAV NAb testing is a streamlined process. All blood samples for AAV NAb testing are sent abroad (USA or Europe) (Fig. 2f). It takes 1 –2 weeks for the results to be received by the neurologist (Supplementary Table 1). Gene therapy must be infused within 1 month of NAb testing otherwise the test needs to be repeated (this is the current experience with gene replacement therapy used to treat SMA). The requirements of health authorities regarding blood shipment abroad include (but are not limited to):

• The laboratory or manufacturer needs to take care of the logistics, including assuming full responsibility for the shipment of the samples and completion of all the health authority paperwork
• A declaration of a medical sample for testing is required
• Approval of laboratories via a central laboratory is required

### Preparing for DMD gene therapy

#### Insights on the readiness for gene therapy

Education on DMD and gene therapy for medical faculty, nursing and support staff; staff training in preparation and safe administration and post-infusion follow-up of gene therapy; rationale for patient selection and patient risk stratification; information on DMD and gene therapy for patients / families; management approval of gene therapy; early accessible genetic testing and resolution of reimbursement and cost issues are considered the top priorities to improve readiness for the introduction of gene therapy for DMD in the UAE and Kuwait. Based on our survey, 88% (*n* = 7) of the experts already have experience with other gene therapies (Supplementary Table 1).

Facilities and resources for the preparation and safe administration of gene therapy, including logistical set-up for the supply and storage of gene therapies are also required.

### Insights on educational needs: Who should learn what?

Each person involved in the management of a patient with DMD should be well informed about the disease and the management thereof. Table 3 illustrates the educational needs of clinicians, families and caregivers, and payers.

**Table 3:** Educational needs of people involved in the care of patients with DMD

General	Specialist	Nurses	Pharmacists	Caregivers/	Payers/
pediatricians	physicians	parents	management
DMD disease burden and prognosis	✓	✓	✓	✓
Signs &symptoms of DMD for referral	✓	✓	✓	✓
Diagnosis of DMD	✓	✓
DMD Management and Care	✓	✓	✓	✓	✓
Science behind gene therapy	✓	✓	✓	✓	✓	✓
Efficacy &safety of gene therapy	✓	✓	✓	✓	✓
Preparation &administration of gene therapy	✓	✓

Clinicians should be well trained on trial and safety-like clinical data of gene therapies indicated for DMD. It is further important that families and clinicians alike have a clear understanding of the risk-benefit ratio and cost of treatment, as well as realistic expectations of gene therapy, i.e., that gene therapy is not a cure and that patients would still require MDT care to manage their condition.

In addition to the specific training needs, it is also important that other stakeholders such as rehabilitation teams, procurement departments, health insurers and Revenue Cycle Management (RCM) are educated on the clinical benefits and cost-effectiveness of gene therapy.

### Practical considerations of gene therapy in DMD patients

**Fig. 4:**
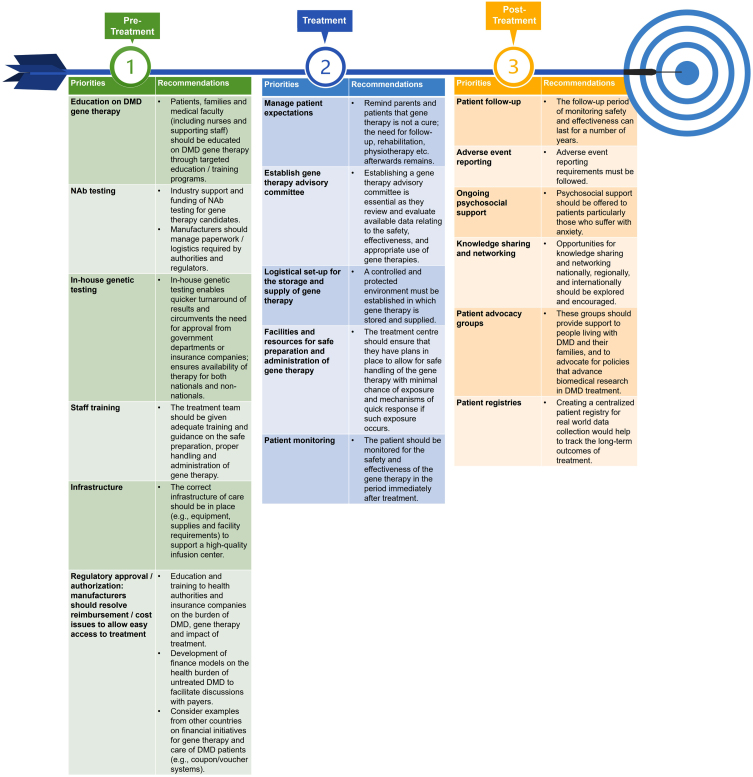
Priorities and recommendations for DMD gene therapy.

Figure 4 illustrates the priorities and recommendations for DMD gene therapy.

## CONCLUSION

There are several challenges facing the management of DMD patients in the UAE and Kuwait and most likely other countries within the Middle East with less resourced healthcare systems. This paper offers insights based on expert opinion on how to improve on current practice. Furthermore, it highlights gene therapy as a promising approach to treating DMD in the future. Efforts to successfully implement AAV/ micro-dystrophin gene therapy in the UAE and Kuwait will require careful planning, education, capacity building and prioritization of core initiatives. These include setting up a patient registry, hospital collaborations, formulating a unified care approach and local guidelines, establishing training programs for paediatricians and primary healthcare physicians and assistance in genetic confirmation tests. Establishing a timeline for the action plan would be heavily dependent on the priorities, readiness and collaborative work between many different entities to set up a country plan. Such entities would include Ministries of Health, healthcare authorities, hospitals, physicians, societies etc. Complexities of healthcare systems, such as that in the UAE, which is mainly private driven, could pose an additional challenge in the implementation of gene therapy according to a defined timeline.

The recommendations provided are intended to assist HCPs, payers and regulators to better understand the current DMD management landscape in the UAE and Kuwait and to assess the potential opportunities and challenges for gene therapy. Given the expectedly high genetic disease burden in the Middle East, it is important to streamline DMD patient diagnosis, management, and treatment for better outcomes in this region, and for overall understanding of the natural history of the disease and efficacy of DMD gene therapy.

## LIMITATIONS

There were a small number of respondents to the pre-meeting survey which could be considered a limitation however, these experts are directly involved in the regular care of patients with DMD in the UAE and Kuwait and were selected as they are the physicians responsible for running genetic / neuromuscular services. Patients in primary and secondary care levels are referred to these physicians. As such, although there were few respondents, the quality of their responses (based on their extensive experience) is high.

## Summary points

•DMD is a severe, progressive genetic childhood disorder that results in functional decline, loss of ambulation and early death due to cardiac or respiratory failure.

•This paper reviews the current practices and challenges in the management of DMD in the UAE and Kuwait and how to prepare for gene therapy, based upon local expert opinions and recommendations.

•Awareness issues for referral are similar in the UAE and Kuwait, and for nationals compared to non-nationals.

•Education to increase awareness of DMD is the mainstay of achieving an earlier diagnosis. As such, there is a great need for awareness and educational campaigns for both HCPs and the public.

•The value and simplicity of CK testing should be emphasized.

•In the UAE: establishing early accessible in-country genetic testing for DMD diagnosis is crucial to collect phenotype and genotype data.

•Support is needed to establish MDT clinics that provide universal care (irrespective of insurance coverage) to all DMD patients.

•Training to unify DMD patient assessment is needed especially for physiatrists and physiotherapists

•Ideally, DMD management should be in a centre of excellence and involve a multidisciplinary approach.

•The top three collaborative activities considered to be of value include research, education, and awareness / advocacy.

•Financial models are needed for reimbursement and access discussions with health authorities and insurance companies to educate them on the health economic burden of untreated DMD patients and the direct and indirect cost savings derived from early treatment.

•Gene therapy offers a promising approach to treating DMD however careful planning, education, capacity building and prioritization of core initiatives are required.

## Supplementary Material

Supplementary Material
