# Supplementary material for: Gene Therapy for Duchenne Muscular Dystrophy: Unlocking the Opportunities in Countries in the Middle East and Beyond
Source: J Neuromuscul Dis. 2022 Nov 8;9(6):787–801. doi: 10.3233/JND-221528 (PMC9697043; doi:10.3233/JND-221528)
Supplement: Supplementary Material [file jnd-9-jnd221528-s001.docx]

**Supplementary Table 1 Gene therapy in Duchenne Muscular Dystrophy: Pre-meeting Survey and Results**

| **DMD in the United Arab Emirates and Kuwait** |  | | | | | | | | |
| --- | --- | --- | --- | --- | --- | --- | --- | --- | --- |
|  | How many new DMD patients do you see each year? | **0 - 1** | **2 - 5** | **6 - 10** | **>10** |  |  |  |  |
|  | N=9 | 11% | 78% | 11% |  |  |  |  |  |
|  | How many DMD patients do you see for follow-up each month? | **0 - 1** | **2 - 5** | **6 - 10** | **>10** |  |  |  |  |
|  | N=9 | 22% | 56% | 22% |  |  |  |  |  |
|  | What proportion of your DMD patients are nationals versus non-nationals? | **More nationals** | **50/50** | **More non-nationals** | **N/A** |  |  |  |  |
|  | N=9 | 33% | 22% | 33% | 11% |  |  |  |  |
|  | Is there a local or regional ‘center of excellence’ for DMD in your country? | **Yes** | **No** | **N/A** |  |  |  |  |  |
|  | N=9 | 11% | 78% | 11% |  |  |  |  |  |
|  | Are you aware of institutions in your country being involved in past or ongoing clinical trials related to neuromuscular disorders or DMD? | **Yes** | **No** | **N/A** |  |  |  |  |  |
|  | N=9 |  | 89% | 11% |  |  |  |  |  |
|  | Are you aware of any planned or ongoing screening programs for DMD or other neuromuscular disorders in your country? | **Yes** | **No** | **N/A** |  |  |  |  |  |
|  | N=9 | 11% | 89% |  |  |  |  |  |  |
|  | Do you collect genetic and clinical information either formally or informally in a data repository/patient registry? | **Yes** | **No** | **N/A** |  |  |  |  |  |
|  | N=9 | 33% | 56% | 11% |  |  |  |  |  |
| **DMD Referral** | Who are the main sources of referrals for patients with DMD? | **Pediatricians** | **Family medicine physicians** | **Family/care givers/ teachers** | **Other** |  |  |  |  |
|  | N=8  *More than one answer allowed* | 67% | 17% | 8% | 8% |  |  |  |  |
|  | What are the common motives for DMD patient referral? | **Muscle weakness / motor symptoms** | **Family history / genetic testing** | **Elevated serum creatine kinase** | **Neurologic / non-motor symptoms** |  |  |  |  |
|  | N=8  *More than one answer allowed* | 35% | 26% | 30% | 9% |  |  |  |  |
|  | What is the age range of DMD patients referred to you? | **0-3yrs** | **3-6yrs** | **6-9yrs** | **>10yrs** |  |  |  |  |
|  | N=8 |  | 75% | 25% |  |  |  |  |  |
|  | Is any tool or material available for red flag signs or symptoms for DMD to improve the referral? | **Yes** | **No** |  |  |  |  |  |  |
|  | N=8 | 12% | 88% |  |  |  |  |  |  |
| **DMD Diagnosis** | What is the average wait-time between referral and diagnosis? | **0-1yr** | **2-5yrs** | **>5yrs** | **I don’t know** |  |  |  |  |
|  | N=8 | 88% |  |  | 12% |  |  |  |  |
|  | How many healthcare professionals do your patients visit before receiving a diagnosis of DMD? | **1-2** | **3-5** | **>5** |  |  |  |  |  |
|  | N=8 | 38% | 62% |  |  |  |  |  |  |
|  | What is the average age of your DMD patients at time of diagnosis? | **0-3** | **3-6yrs** | **6-9yrs** | **>10yrs** |  |  |  |  |
|  | N=8 |  | 100% |  |  |  |  |  |  |
|  | Does your institution use a standardized algorithm for diagnosis of DMD? | **Yes** | **No** |  |  |  |  |  |  |
|  | N=8 | 75% | 25% |  |  |  |  |  |  |
|  | Which diagnostic tests for DMD are available within your institution, or can be accessed locally, or need to be sent abroad? | **DMD PCR genetic studies** | **DMD MLPA genetic studies** | **DMD mutation specific genetic studies** | **Other** |  |  |  |  |
|  | Sent abroad  Locally  Institution  N/A | 38% | 63% | 63% | 29% |  |  |  |  |
|  |  | 25% | 12% | 12% |  |  |  |  |  |
|  |  | 25% | 25% | 25% | 42% |  |  |  |  |
|  |  | 12% |  |  | 29% |  |  |  |  |
| **DMD Treatment** | Do you routinely follow guidelines on standards of care for patients with DMD in your institution? | **Yes** | **No** | **N/A** |  |  |  |  |  |
|  | N=8 | 88% |  | 12% |  |  |  |  |  |
|  | What percentage of your DMD patients receive corticosteroids? | **<25%** | **25-50%** | **50-75%** | **>75%** |  |  |  |  |
|  | N=8 |  | 25% | 13% | 62% |  |  |  |  |
|  | At what age do your patients typically receive corticosteroids? | **0-4yrs** | **4-6yrs** | **6-10yrs** | **>10yrs** |  |  |  |  |
|  | N=8 | 12% | 88% |  |  |  |  |  |  |
|  | Which corticosteroids do you typically prescribe for DMD? | **prednisone** | **deflazacort** | **other** |  |  |  |  |  |
|  | N=8 | 38% | 50% | 12% |  |  |  |  |  |
|  | Do you have access to exon skipping therapies in your country to treat DMD? | **Yes** | **No** |  |  |  |  |  |  |
|  | N=8 | 100% |  |  |  |  |  |  |  |
|  | Do patients/caregivers/physicians face challenges with current treatment options? | **Yes** | **No** |  |  |  |  |  |  |
|  | N=8 | 62% | 38% |  |  |  |  |  |  |
|  | Who pays for medical treatment of DMD? | **Private insurance** | **Publicly funded for nationals and non-nationals** | **Publicly funded for nationals only** | **Patient’s family** | **Other** |  |  |  |
|  | N=8 | 50% | 12% | 38% |  |  |  |  |  |
|  | How often do your DMD patients return for follow-up/monitoring? | **Every month** | **3-6 months** | **6-12 months** | **yearly** | **1-2years** |  |  |  |
|  | N=8 | 12% | 88% |  |  |  |  |  |  |
| **Multidisciplinary Care** | In your hospital, which healthcare professionals are part of multidisciplinary team management of your patients with DMD? | **Neuromuscular specialist** | **Pediatrician** | **Cardiologist** | **Pulmonologist** | **Rehabilitation specialist** | **Dietician** | **Genetic councellor** | **Other** |
|  | N=8  *More than one answer allowed*. | 15% | 8% | 18% | 10% | 18% | 15% | 10% | 6% |
|  | Are there any hurdles in transitioning patients with DMD from pediatric to adult care? | **Yes** | **No** | **N/A** |  |  |  |  |  |
|  | N=8 | 63% | 25% | 12% |  |  |  |  |  |
| **Support and Ongoing Care** | Are there patient association groups for DMD in your country? | **Yes** | **No** |  |  |  |  |  |  |
|  | N=8 | 38% | 62% |  |  |  |  |  |  |
|  | What role and services do you think patient association groups for DMD should provide? | **Emotional support for patients and families** | **Practical daily living support** | **Awareness and education on DMD** | **Information on medical care and care team** | **Advice on access and reimbursement of medical care** | **Information on clinical trials** |  |  |
|  | N=8  *More than one answer allowed.* | 100% | 88% | 100% | 75% | 62% | 62% |  |  |
| **Gene Therapy** | Is there a gene therapy committee in your country? | **Yes** | **No** | **I don’t know** |  |  |  |  |  |
|  | N=8 | 25% | 37.5% | 37.5% |  |  |  |  |  |
|  | Do you have experience with other gene therapies in your country? | **Yes** | **No** |  |  |  |  |  |  |
|  | N=8 | 88% | 12% |  |  |  |  |  |  |
|  | For many of the emerging gene therapies, prior to receiving therapy, candidates must receive a negative test result for antibodies against AAV (adenovirus-associated vector). Where is the neutralizing antibody testing (NAb) done? | **Locally** | **Abroad** |  |  |  |  |  |  |
|  | N=8 |  | 100% |  |  |  |  |  |  |
|  | Are blood samples allowed to be sent outside the country for testing purposes? | **Yes** | **No** | **I don’t know** |  |  |  |  |  |
|  | N=8 | 88% |  | 12% |  |  |  |  |  |
|  | How long does it take to get the NAb test results? | **1 week** | **2 weeks** | **3 weeks** | **1 month** | **Other** | **N/A** |  |  |
|  | N=8 | 50% | 25% |  |  | 12.5% | 12.5% |  |  |
|  | Are there any hurdles associated with NAb testing? | **Yes** | **No** |  |  |  |  |  |  |
|  | N=8 | 12% | 88% |  |  |  |  |  |  |
|  | Do you anticipate your hospital to be a gene therapy infusion center or referring center? | **Infusion center** | **Referring center** |  |  |  |  |  |  |
|  | N=8 | 75% | 25% |  |  |  |  |  |  |
|  | Where is there opportunity for collaboration in the fields of DMD and gene therapy? | **Between hospitals** | **Between countries in the region** | **Internationally** |  |  |  |  |  |
|  | N=8  *More than one answer allowed.* | 42% | 26% | 32% |  |  |  |  |  |
|  | What types of collaborative activities would be of value? | **Testing / diagnosis** | **Treatment** | **Education** | **Research** | **Awareness / advocacy** |  |  |  |
|  | N=8  *More than one answer allowed.* | 16% | 19% | 22% | 22% | 22% |  |  |  |

N= number of respondents

% ~ the percentage of respondents who selected the relevant answer to the question
